# From Nodal Metastasis to Oncologic Recurrence in Tongue and Oral Floor Squamous Cell Carcinoma: Distinct Failure Patterns and Clinicopathological Risk Profiles

**DOI:** 10.1002/hed.70349

**Published:** 2026-06-09

**Authors:** Jannik Ketschau, Jonathan Mohr, Alex Grabenhorst, Hannes Singer, Helena Kram, Cornelius Leopold, Nils Krautkremer, Katharina Pippich, Klaus‐Dietrich Wolff, Lucas M. Ritschl

**Affiliations:** ^1^ Department of Oral and Maxillofacial Surgery, TUM University Hospital Klinikum Rechts der Isar, School of Medicine and Health Technical University of Munich Munich Germany

**Keywords:** Kaplan–Meier analysis, lymph node ratio, neck dissection, oncologic recurrence, Oral squamous cell carcinoma

## Abstract

**Background:**

Locoregional recurrence remains a clinically relevant problem in oral squamous cell carcinoma (OSCC). Whether local, ipsilateral nodal, and contralateral nodal failure represent distinct disease processes with different clinicopathological associations has not been systematically evaluated.

**Methods:**

This retrospective single‐center study included patients only with primary OSCC of the tongue or floor of mouth undergoing surgical resection and neck dissection (2011–2021; *n* = 438). Oncologic recurrence patterns were evaluated in patients with at least six months of follow‐up (*n* = 363) using multivariable Cox regression adjusted for adjuvant therapy and Kaplan–Meier analyses.

**Results:**

Nodal involvement at primary surgery was associated with depth of invasion, bone infiltration, and posterior tongue subsites. In the follow‐up cohort, 120 patients (33.1%) developed recurrence. Local recurrence (18.5%) was independently associated with extranodal extension (HR 1.88, 95% CI 1.04–3.40; *p* = 0.037). Ipsilateral nodal recurrence (11.0%) was associated with lymph node ratio (LNR) > 0.1 versus LNR = 0 (HR 4.90, 95% CI 1.76–13.63; *p* = 0.002). Contralateral nodal recurrence (8.5%) was not predicted by any evaluated parameter. Patients with LNR > 0.1 experienced recurrence after a median of 19.2 versus 103.0–112.4 months (*p* < 0.001).

**Conclusion:**

Local and ipsilateral recurrences showed distinct associations with clinicopathological factors, while contralateral recurrence remained poorly predicted by routine parameters. Lymph node ratio was associated with ipsilateral recurrence risk and shorter event‐free survival, supporting its use as a potential stratification marker for risk‐adapted surveillance.

**Abbreviations**
ASA: American society of anesthesiologists (physical status classification)
AUC: area under the curve
BMI: body mass index
CI: confidence interval
cN0: clinically node‐negative
DOI: depth of invasion
ENE: extranodal extension
HR: hazard ratio
IQR: interquartile range
KM: Kaplan–Meier
LNR: Lymph node ratio
ND: neck dissection
OSCC: oral squamous cell carcinoma
pN: pathological nodal stage
pT: pathological tumor stage
ROC: receiver operating characteristic
SAP: systems, applications, and products in data processing
SD: standard deviation

## 1 Introduction

Oral squamous cell carcinoma (OSCC) is commonly treated by surgical resection of the primary tumor combined with elective management of the cervical lymph nodes [1, 2, 3]. Despite advances in surgical techniques and adjuvant therapy, recurrence remains a clinically relevant problem and is associated with impaired disease control and survival [4, 5].

In patients with clinically node‐negative (cN0) OSCC, selective neck dissection (ND) of levels I–III represents a widely accepted standard approach [6, 7]. However, regional recurrences may still occur after primary treatment, either within the ipsilateral neck or at the contralateral side. Such events are of clinical relevance, as they often require additional treatment and may substantially affect subsequent oncologic outcomes [8, 9].

Regional nodal recurrences in OSCC predominantly occur on the ipsilateral side and most frequently involve level II lymph nodes. Contralateral nodal recurrence is less common but has been reported particularly in tumors of the tongue and floor of mouth, especially in more advanced disease or in lesions with midline proximity [10, 11, 12]. Metastatic involvement or recurrence in cervical lymph node levels beyond I–III appears to be uncommon, but the associated risk factors and the influence of the initial neck dissection extent remain insufficiently defined [13, 14]. While nodal parameters such as lymph node ratio (LNR) and extranodal extension have been associated with recurrence risk in OSCC, it remains unclear whether local, ipsilateral nodal, and contralateral nodal failure share common pathological drivers or represent distinct disease processes—and whether these parameters can stratify not only recurrence risk but also its timing [15, 16].

We hypothesized that pathological findings at primary surgery differentially predict distinct patterns of subsequent failure, and that local, ipsilateral nodal, and contralateral nodal recurrences represent mechanistically distinct entities driven by different clinicopathological factors. To test this hypothesis, we analyzed predictors of nodal involvement at primary surgery and characterized local and regional recurrence patterns using multivariable Cox regression and time‐to‐event analyses in a cohort of 438 patients with OSCC of the tongue and floor of mouth.

## 2 Materials and Methods

### 2.1 Ethical Considerations, Study Design and Patient Identification

This retrospective cohort study included patients treated for primary OSCC at our institution between 2011 and 2021. Patients were initially identified via diagnosis code‐based queries in the institutional hospital information system (SAP). Case identification was subsequently verified using the electronic medical record and the digital patient archive. Clinical, surgical, pathological, radiological, and follow‐up data were extracted from the digital patient archive and pseudonymized prior to analysis. Due to the retrospective design and evolving documentation standards over the study period, some variables were not available for all patients.

### 2.2 Two‐Stage Study Population

Analyses were performed in two stages: Stage 1 (primary treatment cohort) included all patients with primary OSCC only of the tongue or floor of mouth who underwent primary surgical resection with curative intent. Exclusion criteria were recurrences and second primary tumors. This stage was used to describe baseline clinicopathologic characteristics and primary treatment patterns. Stage 2 (follow‐up cohort for recurrence analyses) was restricted to patients with tongue or floor‐of‐mouth OSCC who had either (i) a minimum follow‐up duration of ≥ 6 months or (ii) developed an event (recurrence/malignancy) before 6 months. This restriction was applied to reduce outcome misclassification due to insufficient surveillance time.

Patients without any documented follow‐up were excluded from time‐to‐event analyses.

### 2.3 Definitions and Treatment Variables

The index side was defined as the side harboring the main tumor burden. In tumors crossing the midline, the side with dominant tumor extension was defined as the index side. Neck dissection strategy was categorized by laterality only and was defined as ipsilateral (performed on the index side only) or bilateral (performed on both sides). The decision for bilateral neck dissection was based on clinico‐radiological assessment of contralateral nodal risk, including tumor size, midline proximity, nodal status (morphology, nodal size, internal architecture, signs suggestive of extranodal extension). Based on these interdisciplinary parameters, the decision reflected institutional practice and national guideline over the study period [17].

### 2.4 Endpoints

Recurrence endpoints were primarily analyzed as time‐to‐event outcomes. Binary recurrence variables were used descriptively to report recurrence patterns and enable comparison with prior literature

- Overall oncologic recurrence (yes/no)
- Lymph node recurrence overall (yes/no) (ipsilateral and/or contralateral)
- Ipsilateral lymph node recurrence (yes/no)
- Contralateral lymph node recurrence (yes/no) (relative to the index side)

If available, nodal level ≥ IV recurrence was reported descriptively, but was not used as a primary multivariable endpoint, as event counts were low.

Time‐to‐event was calculated from the date of primary surgery to the first occurrence of the respective endpoint (recurrent malignancy, overall lymph node recurrence, ipsilateral lymph node recurrence, or contralateral lymph node recurrence). Patients without an event were censored at the date of last documented follow‐up.

### 2.5 Variables and Data Handling

Collected clinicopathological variables included patient demographics (e.g., age), primary tumor site (tongue vs. floor of mouth), pathological T stage, depth of invasion (DOI in mm), bone invasion, extranodal extension (ENE), number of removed lymph nodes, number of positive lymph nodes, and lymph node ratio (LNR) (including categorical lymph node ratio groups) [18]. The LNR was analyzed as a categorical variable (0, > 0–0.1, > 0.1) and the initial lymph node involvement was categorized in level I–III and level ≥ IV to improve interpretability and model stability. Smoking, alcohol consumption, diabetes, and ASA score were extracted when available. Adjuvant therapy (radiotherapy or chemoradiotherapy) was documented and included as a covariate in all multivariable analyses.

Missing data were handled using model‐specific complete‐case analyses. Thus, the number of included cases varied across models depending on variable availability. Multiple imputation was not applied, as the proportion of missing data was low and missing values were considered to occur at random due to evolving documentation standards rather than systematically. Complete case analysis is therefore unlikely to have introduced substantial bias.

### 2.6 Statistical Analysis

Descriptive statistics were used to summarize baseline characteristics. Continuous variables were summarized using mean ± standard deviation or median (IQR) and compared using the Mann–Whitney U test (two groups) or Kruskal–Wallis test (more than two groups), as appropriate. Categorical variables were compared using chi‐square tests or Fisher's exact test where applicable.

For each endpoint, variables were selected for multivariable analysis based on clinical relevance, and models were intentionally kept parsimonious to minimize overfitting given the number of available events.

Time‐to‐event outcomes were analyzed using Kaplan–Meier estimates and compared using the log‐rank test. In addition, multivariable Cox proportional hazards regression was performed for each endpoint to evaluate independent associations with event timing. For categorical variables entered as dummy‐coded groups (e.g., LNR categories), the global *p*‐value reflects the overall significance of the variable across all categories, derived from the likelihood ratio test. The proportional hazards assumption was assessed using log‐minus‐log plots and/or time‐dependent covariate checks (e.g., visual inspection of Schoenfeld‐type diagnostics as available in SPSS workflows).

Given the retrospective and non‐randomized nature of ND strategy, all comparisons of ipsilateral versus bilateral ND were interpreted as associative rather than causal and discussed with attention to potential confounding by indication.

All analyses were performed using IBM SPSS Statistics (version 29). A two‐sided *p*‐value < 0.05 was considered statistically significant.

## 3 Results

### 3.1 Results Primary Treatment Cohort

**TABLE 1:** Baseline patient characteristics in primary treatment group.

Patient Characteristics	Total (*n* = 438)
Gender	
Female	148 (33.8%)
Male	290 (66.2%)
Age	60.2 (53.0–68.3)
Height (m)	1.73 ± 0.10
Weight (kg)	73.82 ± 15.30
BMI	24.15 ± 4.81
ASA	
I	32 (7.3%)
II	325 (74.2%)
III	80 (18.3%)
IV	1 (0.2%)
Smoking	
No	172 (39.3%)
Yes	266 (60.7%)
Alcohol	
No	298 (68%)
Yes	140 (32%)
Tumor characteristics	
Location	
Floor of the mouth	251 (57.3%)
Tongue	187 (42.7%)
Tumor subsite – tongue	
Anterior third	31 (16.4%)
Middle third	92 (49.1%)
Posterior third	50 (26.6%)
Multiple subsites	14 (7.3%)
Bone infiltration	
No	371 (85.3%)
Yes	64 (14.7%)
T‐stage (pT)	
T1	180 (41%)
T2	132 (30.1%)
T3	63 (14.4%)
T4	63 (14.4%)
Depth of invasion (mm)	6.0 (3–11)
N‐stage (pN)	
N0	282 (64.4%)
N1	54 (12.3%)
N2	88 (20.1%)
N3	13 (3.0%)
Pn‐stadium	
Pn0	322 (73.5%)
Pn1	115 (26.3%)
Pn2	1 (0.2%)
Resection margin status	
R0	423 (96.6%)
R1	15 (3.4%)
Surgical treatment	
Neck dissection	
Ipsilateral	196 (44.7%)
Bilateral	242 (55.3%)
Number of lymph nodes removed	33.0 (22.0–49.0)
Lymph node levels (histopathology)	
Lymph node ratio (positive/total)	0.0 (0.0–0.3)
Number of positive lymph nodes	0.0 (0.0–1.0)
Histopathology pos. lymph nodes	
No	284 (64.8%)
Yes	154 (35.1%)
Extranodal extension	
No	379 (86.5%)
Yes	59 (13.5%)
Level Ia metastasis	
No	405 (92.5%)
Yes	33 (7.5%)
Level Ib metastasis	
No	376 (85.8%)
Yes	62 (14.2%)
Level II and III metastasis	
No	325 (74.2%)
Yes	113 (25.8%)

*Note:* Continuous variables are presented as mean ± standard deviation (SD) or median with interquartile range (IQR), as appropriate. Categorical variables are reported as counts and percentages.

### 3.2 Baseline Characteristics of Primary Treatment Cohort

A total of 438 patients with primary OSCC were included in this study (Figure 1). The cohort comprised 290 male (66.2%) and 148 female patients (33.8%) with a median age of 60.2 (IQR 53.0–68.3) years. The majority of patients were classified as ASA class II (74.2%), while 18.5% were ASA III–IV. Smoking was documented in 60.7% of patients, and regular alcohol consumption in 32.0% (Table 1).

**FIGURE 1:**
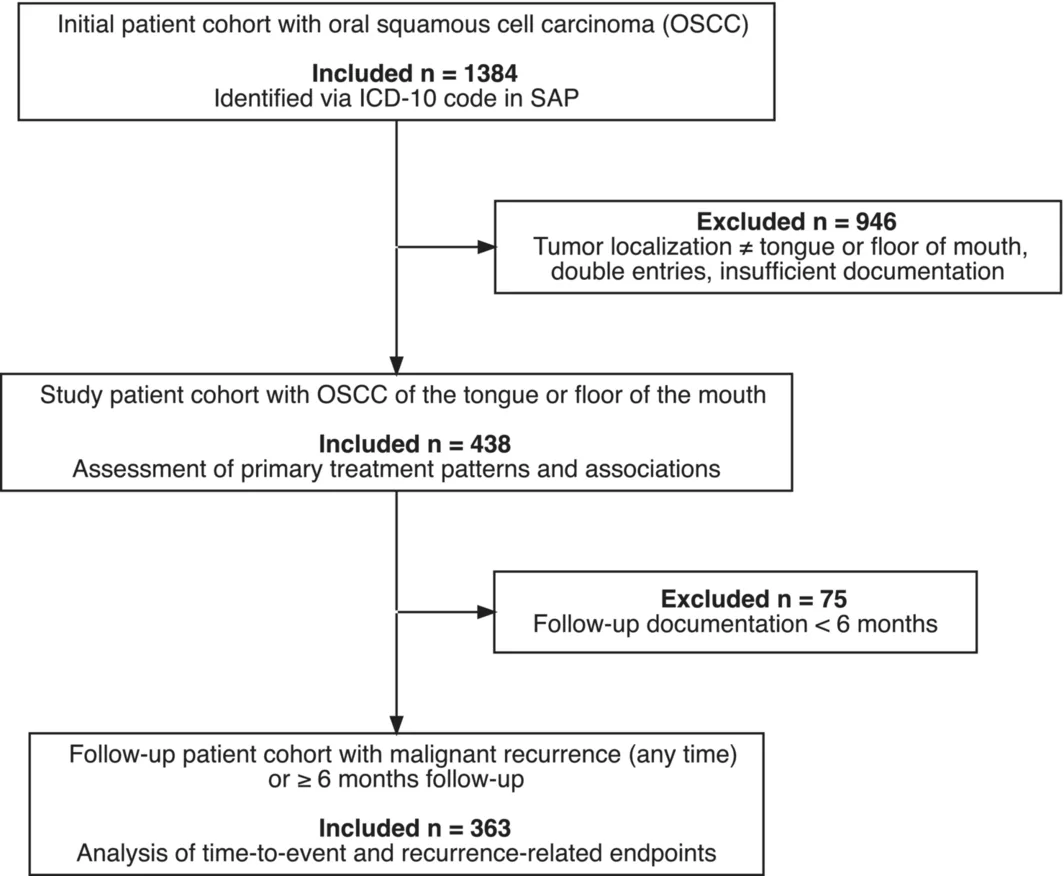
Flowchart of patient selection. Study flowchart illustrating patient selection and cohort definition.

The primary tumors were located in the floor of mouth in 251 patients (57.3%) and in the tongue in 187 patients (42.7%). Seventy‐three cases (29.1%) of oral floor carcinomas were considered unilateral. Bone infiltration was present in 64 patients (14.7%). Among tongue carcinomas, the most frequent subsites were the middle third (49.1%) and posterior third (26.6%), whereas 7.3% involved multiple subsites.

Ipsilateral neck dissection was performed in 196 patients (44.7%), while 242 patients (55.3%) underwent bilateral neck dissection. Histopathological examination revealed lymph node metastases in 154 patients (35.1%), and extranodal extension in 13.5%. The median number of removed lymph nodes was 33.0 (IQR 22.0–49.0), with a median LNR of 0.0 (IQR 0.0–0.3).

### 3.3 Tumor Biology and Nodal Metastasis in Primary Treatment Cohort

Depth of invasion was strongly associated with nodal metastasis. Patients with histopathologically confirmed lymph node involvement (pN+) showed significantly greater DOI compared with pN‐negative patients (*p* < 0.001). Receiver operating characteristic analysis demonstrated a moderate discriminative performance of DOI for predicting nodal metastasis (AUC = 0.685), with an optimal cutoff around 5–6 mm (Youden's index 0.322, sensitivity 73.5%, specificity 58.7%).

Bone infiltration was also significantly associated with nodal involvement. Patients with bone invasion had higher pN+ rates and showed a significantly increased nodal tumor burden, reflected by both the number of positive lymph nodes and LNR (all *p* < 0.001).

While overall tumor localization (tongue vs. floor of mouth) was not associated with nodal metastasis (*p* = 0.859), a clear anatomical gradient was observed within tongue carcinomas. Posterior and overlapping tongue subsites demonstrated significantly higher rates of nodal involvement compared with anterior and middle subsites (*p* = 0.003). This pattern was consistent for both the absolute number of positive lymph nodes and LNR, indicating increasing nodal tumor burden with more posterior tumor location (Table S1).

### 3.4 Results Follow‐Up Cohort

**TABLE 2:** Oncologic recurrence patterns in follow‐up cohort.

Cohort size	*n* = 363 (100%)
Oncologic recurrence (local, regional or combination)	120 (33.1%)
Type of recurrence (*Percentages refer to cohort size*, *n = 363*)	
Local	67 (18.5%)
Regional recurrence ipsilateral	40 (11.0%)
Regional recurrence contralateral	31 (8.5%)
Local recurrence	67 (18.5%)
Lymph node recurrence (any side)	59 (16.3%)
Regional recurrence – ipsilateral	40 (11.0%)
Ipsilateral regional recurrence—lymph node level (*Percentages refer to ipsilateral lymph node recurrences*, *n = 40*)	
1a	2 (5.0%)
1b	3 (7.5%)
2	8 (20.0%)
3	2 (5.0%)
4	4 (10.0%)
5	10 (25.0%)
Combined	11 (27.5%)
Ipsilateral regional recurrence level ≥ IV	25 (6.9%)
Regional recurrence – contralateral	31 (8.5%)
Contralateral regional recurrence – lymph node level (*Percentages refer to contralateral lymph node recurrences*, *n = 31*)	
1a	2 (6.5%)
1b	7 (22.6%)
2	3 (9.7%)
3	2 (6.5%)
4	4 (12.9%)
5	2 (6.5%)
Combined	11 (35.5%)
Contralateral regional recurrence level ≥ IV	17 (4.7%)
Follow‐up (months)	45.1 (22.0–65.9)

*Note:* Data are presented as counts and percentages unless otherwise specified. Follow‐up time is reported as median with interquartile range (IQR).

### 3.5 Follow‐Up and Oncologic Recurrence

Analyses were restricted to patients with at least 6 months of follow‐up or an oncologic event (*n* = 363). Median follow‐up duration was 45.1 months (IQR 22.0–65.9). Overall, 120 patients (33.1%) developed an oncologic recurrence, including local recurrence in 67 patients (18.5%), ipsilateral regional recurrence in 40 patients (11.0%), and contralateral regional recurrence in 31 patients (8.5%) (Table 2).

### 3.6 Overall Oncologic Recurrence

In multivariable Cox proportional hazards analysis adjusted for adjuvant therapy and neck dissection laterality, lymph node ratio (LNR) was independently associated with overall recurrence risk (global *p* = 0.030). Patients with LNR > 0.1 showed a significantly increased hazard of recurrence compared with patients with LNR = 0 (HR 2.77, 95% CI 1.27–6.05; *p* = 0.010), whereas the intermediate LNR category (> 0–0.1) was not significantly associated with recurrence risk (HR 1.08, 95% CI 0.65–1.79; *p* = 0.766). In addition, adjuvant therapy was associated with shorter event‐free survival (HR 1.81, 95% CI 1.05–3.12; *p* = 0.034). Pathological T stage, ENE, and neck dissection laterality were not significantly associated with overall recurrence.

### 3.7 Local Recurrence

Local recurrence occurred in 67 patients (18.5%). In multivariable Cox regression analysis, extranodal extension was independently associated with an increased hazard of local recurrence (HR 1.88, 95% CI 1.04–3.40; *p* = 0.037). Adjuvant therapy was also associated with local recurrence (HR 2.57, 95% CI 1.24–5.36; *p* = 0.012). Pathological T stage and resection margin status were not significantly associated with local recurrence after adjustment.

### 3.8 Contralateral Regional Lymph Node Recurrence

Contralateral regional recurrence occurred in 31 patients (8.5%). In multivariable Cox regression analysis, no clinicopathological variable, including lymph node ratio, adjuvant therapy, or neck dissection laterality, was significantly associated with contralateral recurrence. Compared with patients with LNR = 0, neither patients with LNR > 0.1 (HR 2.77, 95% CI 0.74–10.35; *p* = 0.130) nor those with LNR > 0–0.1 (HR 0.90, 95% CI 0.35–2.32; *p* = 0.819) demonstrated a significant increase in contralateral recurrence risk. The overall model did not reach statistical significance (*p* = 0.331), likely reflecting limited statistical power due to the low number of events.

### 3.9 Ipsilateral Regional Lymph Node Recurrence

Ipsilateral regional recurrence occurred in 40 patients (11.0%). In Cox regression analysis, the overall LNR variable did not reach conventional significance (global *p* = 0.071); however, patients with LNR > 0.1 showed a significantly increased hazard of ipsilateral recurrence compared with the reference category LNR = 0 (HR 4.90, 95% CI 1.76–13.63; *p* = 0.002), while the intermediate category LNR > 0–0.1 did not differ significantly from the reference (HR 1.25, 95% CI 0.59–2.65; *p* = 0.563). Adjuvant therapy showed a non‐significant trend toward increased recurrence risk (HR 1.98, 95% CI 0.90–4.39; *p* = 0.091), while extranodal extension and neck dissection laterality were not significantly associated.

### 3.10 Regional Recurrence Beyond Level III

Recurrence involving lymph node levels beyond level III (≥ IV) was observed in a subset of patients. Ipsilateral level ≥ IV recurrence occurred in 25 of 363 patients (6.9%), while contralateral level ≥ IV recurrence was observed in 17 patients (4.7%).

Descriptive analysis revealed that level ≥ IV recurrence was not restricted to individuals with advanced nodal disease. Among patients with ipsilateral level ≥ IV recurrence, 56.0% were initially classified as pN0, extranodal extension was present in only 20.0%, and a lymph node ratio > 0.1 in 16.0%. Similarly, among patients with contralateral level ≥ IV recurrence, 64.7% were pN0, extranodal extension was observed in 11.8%, and only 11.8% exhibited a lymph node ratio > 0.1. Both ipsilateral and contralateral level ≥ IV recurrences occurred across all pathological T stages (Table 3). No clear enrichment by primary localization or tongue subsite was observed in descriptive analyses.

Given the limited number of events, no multivariable analyses were performed for these endpoints. These findings are therefore descriptive and should be interpreted with caution.

#### 3.10.1 Clinical Characteristics of Patients With Regional Recurrence Beyond Level III (≥ IV)

**TABLE 3:** Descriptive characteristics of patients who developed ipsilateral or contralateral lymph node recurrence involving cervical levels ≥ IV.

Variable	Ipsilateral ≥ IV recurrence (*n* = 25)	Contralateral ≥ IV recurrence (*n* = 17)
T‐stage (pT)		
pT1	8 (32.0%)	5 (29.4%)
pT2	6 (24.0%)	6 (35.3%)
pT3	4 (16.0%)	4 (23.5%)
pT4	7 (28.0%)	2 (11.8%)
DOI, median (IQR)	4.0 (2.0–13.0)	4.0 (4.0–14.3)
Bone invasion	7 (28.0%)	2 (11.8%)
pN0	14 (56.0%)	11 (64.7%)
pN+	11 (44.0%)	6 (35.3%)
ENE+	5 (20.0%)	2 (11.8%)
Lymph node ratio		
0	14 (56.0%)	11 (64.7%)
> 0–0.1	7 (28.0%)	4 (23.5%)
> 0.1	4 (16.0%)	2 (11.8%)
Adjuvant RTx/RCTx	19 (76.0%)	11 (64.7%)

### 3.11 Kaplan–Meier Survival Analyses

Kaplan–Meier analysis demonstrated significant differences in event‐free survival across lymph node ratio (LNR) categories (log‐rank *p* < 0.001, Figure 2). Patients with LNR > 0.1 showed markedly reduced event‐free survival compared with those with LNR > 0–0.1 and LNR = 0.

**FIGURE 2:**
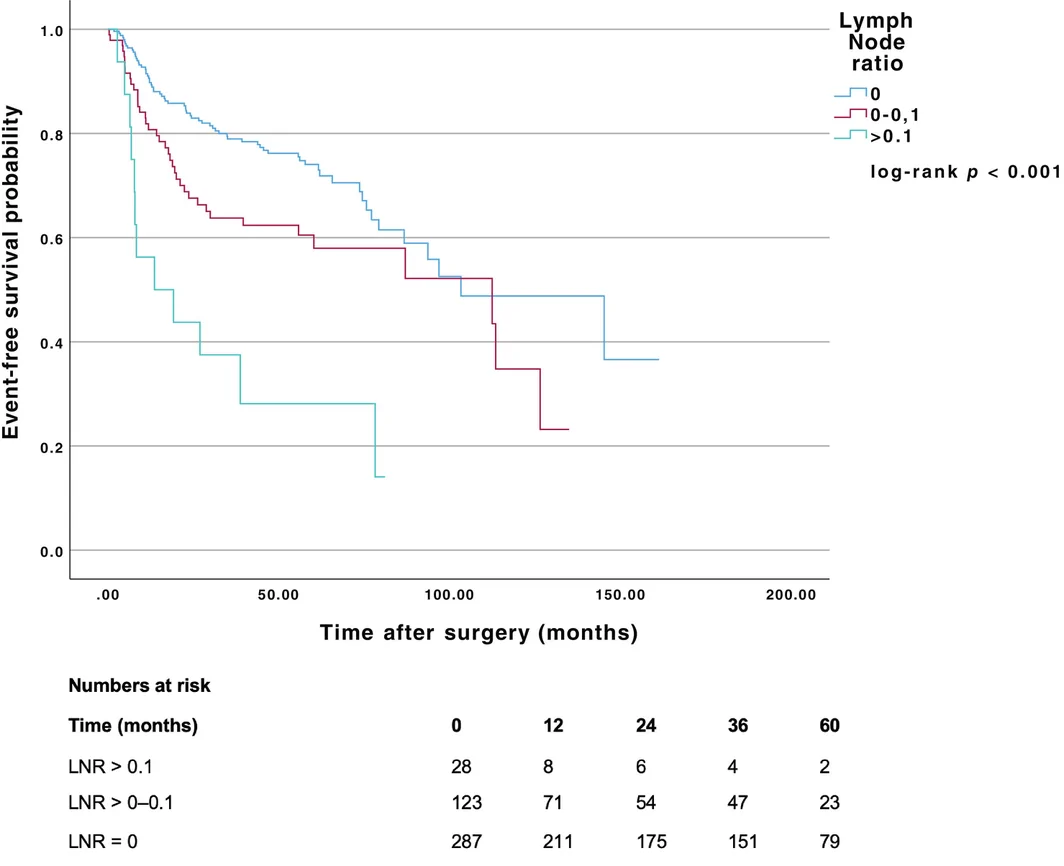
Kaplan–Meier analyses of event free survival. Kaplan–Meier analysis of event‐free survival stratified by lymph node ratio (LNR). Patients with LNR > 0.1 showed significantly shorter event‐free survival compared with lower LNR categories (log‐rank *p* < 0.001). Numbers at risk are shown below the x‐axis at predefined time points. [Color figure can be viewed at wileyonlinelibrary.com]

Median event‐free survival was 19.2 months for patients with LNR > 0.1, compared with 112.4 months for LNR > 0–0.1 and 103.3 months for LNR = 0.

## 4 Discussion

This study examined the full spectrum from nodal metastasis at primary surgery to locoregional recurrence in patients with primary OSCC of the tongue and floor of mouth, with the aim of characterizing distinct local and regional failure patterns and their respective clinicopathological drivers. The present cohort demonstrated an overall oncologic recurrence rate of 33.1%, with 120 of 363 patients developing either local, regional or combined recurrence during a median follow‐up of 45.1 months (IQR 22.0–65.9). Local recurrence represented the most frequent pattern, occurring in 18.5% of cases, even though initial margin‐free resection was achieved in 96.6%. Regional lymph node recurrence was observed in 16.3% of patients, with ipsilateral nodal relapse (11.0%) being more common than contralateral recurrence (8.5%).

### 4.1 Baseline Findings and Immediate Postoperative Outcomes

At primary treatment, pathologically assessed nodal involvement after initial neck dissection was closely associated with features reflecting local tumor extent, including DOI, bone infiltration, and posterior or overlapping tongue subsites. These findings are consistent with established observations that increasing local tumor extension is accompanied by a higher likelihood and extent of regional nodal metastasis [19, 20] with reported DOI thresholds for elective neck dissection ranging from 2 to 4 mm in prior studies, compared with an optimal cutoff of 5–6 mm (AUC = 0.685) in the present cohort [21]. In contrast, tongue versus floor of mouth localization was not independently predictive of nodal involvement. Our findings suggest that nodal risk is better explained by tumor extent and subsite‐specific anatomy, which may account for discrepancies with earlier work reporting higher nodal metastasis rates in tongue carcinomas [22].

### 4.2 Follow‐Up—Lymph Node Involvement

During follow‐up, the prognostic relevance of pathological parameters differed systematically by recurrence pattern, supporting the notion that local, ipsilateral nodal, and contralateral nodal failure represent distinct disease processes with different clinicopathological drivers.

Local recurrence was independently associated with extranodal extension, whereas pathological T stage and resection margin status did not retain independent significance after multivariable adjustment. This dissociation suggests that local failure is predominantly driven by primary tumor aggressiveness and local disease control rather than lymphatic spread—a separation that has been consistently observed across multiple analyses [5, 23], and that emphasizes the continued clinical relevance of achieving adequate surgical margins and primary tumor control.

In contrast, ipsilateral regional recurrence was closely linked to the extent of nodal involvement at primary neck dissection, particularly LNR, underscoring nodal tumor burden as the dominant driver of ipsilateral failure [24, 25]. Although the overall LNR variable did not reach conventional significance at the global level (*p* = 0.071), the specific contrast between LNR > 0.1 and the reference category demonstrated a clinically meaningful and statistically significant association (HR 4.90, 95% CI 1.76–13.63; *p* = 0.002), which is consistent with the categorical nature of the variable and the limited number of events in this subgroup.

This pattern is in line with previous studies identifying LNR [26], ENE [23], and the number of positive lymph nodes [27] as the most robust pathological predictors of regional recurrence in OSCC. The differential predictive value of nodal burden measures for ipsilateral versus local recurrence further supports the concept that these endpoints reflect distinct biological processes.

Contralateral nodal recurrence was the least well predicted failure pattern in this cohort. Neither anatomical localization, nor nodal burden parameters, nor any other evaluated clinicopathological variable independently predicted contralateral recurrence. Although earlier studies have suggested increased contralateral risk for tumors of the floor of mouth [12, 28], particularly in advanced or midline‐adjacent disease, simple anatomical localization did not independently influence contralateral recurrence in our cohort. While LNR > 0.1 showed a directionally increasing but non‐significant association with contralateral recurrence in multivariable Cox analysis, suggesting that extensive nodal tumor burden may contribute to contralateral risk in a subset of patients [5, 12, 29], these associations were weaker and less consistent than those observed for ipsilateral recurrence. Taken together, contralateral recurrence likely reflects a combination of individual lymphatic drainage patterns, tumor extent, and biological variability that is not adequately captured by routine pathological parameters at primary surgery.

Recurrence involving cervical lymph node levels beyond level III (≥ IV) occurred in 25 patients (6.9%) ipsilaterally and 17 patients (4.7%) contralaterally—a rate higher than previously reported in selective neck dissection cohorts [30]. Notably, the majority were classified as pN0 at primary surgery (56.0% and 64.7%, respectively), consistent with the broader observation that a pathologically negative neck does not preclude subsequent regional recurrence [31]. None of the evaluated parameters, including LNR, ENE, pT‐stage, tumor subsite, or primary localization, were significantly associated with level ≥ IV recurrence. This suggests that level ≥ IV failure operates independently of the nodal burden parameters that reliably stratify ipsilateral recurrence within standard levels, and likely reflects lymphatic drainage variability not captured by routine pathological assessment. Conventional risk stratification might therefore be insufficient to identify patients at risk for level ≥ IV failure. These findings remain hypothesis‐generating pending prospective validation.

### 4.3 Follow‐Up—Time‐To‐Event

Time‐to‐event analyses demonstrated a pronounced and clinically meaningful impact of pathological nodal burden on event‐free survival. Patients with a LNR greater than 0.1 experienced recurrence after a median of 19.2 months, compared with 112.4 months for LNR > 0–0.1 and 103.3 months for LNR = 0, a near‐tenfold difference that defines two biologically and temporally distinct patient trajectories. These findings are consistent with previous studies identifying LNR, ENE, and the number of positive lymph nodes as robust independent predictors of early recurrence following primary surgery and neck dissection [26, 27, 29, 32]. The high‐LNR group concentrates recurrence risk within the first two postoperative years, a window during which intensified surveillance could enable earlier detection and timely salvage intervention. These findings provide a pathological basis for risk‐stratified follow‐up protocols that depart from uniform surveillance schedules as recommended in current guidelines [17, 33]. LNR > 0.1, assessable from routine pathology reports without additional cost, represents a straightforward criterion for identifying patients who warrant intensified early postoperative surveillance.

Prior literature suggests that nodal burden parameters primarily predict ipsilateral regional recurrence and overall disease control, whereas contralateral recurrence is more strongly associated with tumor extent and midline involvement rather than nodal metrics alone [11, 24]. This distinction is reflected in our data, where initial nodal burden showed a strong association with adverse time‐to‐event outcomes overall, but a weaker and less consistent relationship with contralateral recurrence, further supporting the notion of distinct failure patterns.

### 4.4 Limitations

This study is limited by its retrospective design and single‐center setting, which may affect follow‐up consistency and generalizability. Adjuvant radiotherapy and chemoradiotherapy were included as covariates in multivariable analyses; however, treatment allocation was non‐randomized and driven by pathological risk factors, introducing confounding by indication that cannot be fully adjusted for in a retrospective setting. The observed associations between adjuvant therapy and recurrence risk should therefore be interpreted with caution. The low number of contralateral and level ≥ IV recurrence events restricts statistical power and limits the ability to identify independent predictors for these failure patterns. Several pathological parameters are inherently correlated, as they originate from the same histopathological assessment, precluding causal interpretation. Consequently, the results should be interpreted as endpoint‐specific associations rather than hierarchical or mechanistic relationships.

## 5 Conclusion

In patients with OSCC of the tongue and floor of mouth, local, ipsilateral nodal, and contralateral nodal recurrences showed different clinicopathological associations and levels of predictability. Nodal tumor burden at primary surgery, particularly LNR, was associated with ipsilateral recurrence and shorter event‐free survival. Contralateral recurrence was not robustly predicted by routine pathological parameters, while level ≥ IV failure occurred in a subset of patients without advanced nodal disease. These findings support pattern‐specific risk assessment after primary treatment but do not allow causal conclusions regarding the optimal laterality or extent of neck dissection.

## Author Contributions

All authors had full access to all the data in the study and had final responsibility for the decision to submit for publication. At least two authors (Jannik Ketschau and Lucas M. Ritschl) assessed and verified the underlying data.

## Funding

The authors have nothing to report.

## Ethics Statement

The study was conducted in accordance with the Declaration of Helsinki. Approval was obtained from the institutional ethics committee prior to data collection (approval number: 2022‐234‐S‐KH).

## Consent

Due to the retrospective nature of the study and the use of anonymized data, the requirement for informed consent was waived.

## Conflicts of Interest

The authors declare no conflicts of interest.

## Supporting information


**Table S1:** Univariable analysis of tumor biology and nodal metastasis in primary treatment cohort.

## Data Availability

The data that support the findings of this study are available on request from the corresponding author. The data are not publicly available due to privacy or ethical restrictions.
