# Supplementary material for: From Nodal Metastasis to Oncologic Recurrence in Tongue and Oral Floor Squamous Cell Carcinoma: Distinct Failure Patterns and Clinicopathological Risk Profiles
Source: Head Neck. 2026 Jun 9;48(12):3244–53. doi: 10.1002/hed.70349 (PMC13755742; doi:10.1002/hed.70349)
Supplement: Supplementary file 1 [file HED-48-3244-s001.docx]

# From Nodal Metastasis to Oncologic Recurrence in Tongue and Oral Floor Squamous Cell Carcinoma: Distinct Failure Patterns and Clinicopathological Risk Profiles

Jannik Ketschau MD ^1^; Jonathan Mohr MD DMD ^1^; Alex Grabenhorst MD DMD ^1^; Hannes Singer MD DMD ^1^; Helena Kram MD ^1^; Cornelius Leopold MD ^1^; Nils Krautkremer MD DMD ^1^; Katharina Pippich MD DMD ^1^; Klaus-Dietrich Wolff MD DMD ^1^; Lucas M. Ritschl MD DMD ^1^

^1^ Department of Oral and Maxillofacial Surgery, TUM University Hospital Klinikum Rechts der Isar, School of Medicine and Health, Technical University of Munich, Germany

**Appendix**

**Corresponding author:**

Jannik Ketschau MD

Department of Oral and Maxillofacial Surgery

TUM University Hospital Klinikum Rechts der Isar

School of Medicine and Health

Technical University of Munich

Ismaninger Strasse 22, D-81675 Munich, Germany

Email: jannik.ketschau@tum.de

**Supplementary Table S1*:* Univariable analysis of tumor biology and nodal metastasis in primary treatment cohort**

| **Outcome** | **Predictor** | **Test** | **Effect** | **p-value** |
| --- | --- | --- | --- | --- |
| pN+ vs pN− | Depth of invasion (DOI, mm) | Mann–Whitney U = 1418; Z = −3.63 | Higher DOI in pN+ patients | **< 0.001*** |
| pN+ (yes/no) | Depth of invasion (DOI, mm) | ROC analysis (AUC = 0.685) | Moderate discrimination |  |
| pN+ | DOI cut-off | Youden index | ≥5.5 mm: Sens 73.5%, Spec 58.7% |  |
| pN+ | Bone invasion (yes/no) | Chi-square | 51.6% pN- vs 32.2% pN+ | **0.003*** |
| Number of positive LNs | Bone invasion (yes/no) | Mann–Whitney | Higher in bone-positive tumors | **< 0.001*** |
| Lymph node ratio | Bone invasion (yes/no) | Mann–Whitney | Higher in bone-positive tumors | **< 0.001*** |
| pN+ | Tumor location (floor of mouth vs tongue) | Chi-square = 0.032 | 34.7% pN- vs 35.5% pN+ | 0.859 |
| pN+ | Tongue subsite | Chi-square = 16.22 | Anterior 26%, middle 29%, posterior 44%, overlapping 79% | **0.003*** |
| Number of positive LNs | Tongue subsite | Kruskal–Wallis H = 21.77 | Monotonic increase across subsites | **< 0.001*** |
| Lymph node ratio | Tongue subsite | Kruskal–Wallis H = 15.85 | Monotonic increase across subsites | **0.001*** |

*Table S1: Univariable analysis of tumor biology and nodal metastasis in primary treatment cohort*

*Continuous variables were compared using the Mann–Whitney U test or Kruskal–Wallis test, as appropriate. Categorical variables were analyzed using Pearson’s chi-square test. ROC analysis was performed to assess the discriminatory performance of depth of invasion for nodal positivity. Significant p values (<0.05) are shown in bold.*
